# Prevalence of complications after the oral rehabilitation with implant-supported hybrid prostheses

**DOI:** 10.4317/medoral.17099

**Published:** 2011-07-15

**Authors:** Júlia Real-Osuna, Nieves Almendros-Marqués, Cosme Gay-Escoda

**Affiliations:** 1 DDS. Resident of the Postgrade of Oral Surgery and Implantology. University of Barcelona Dental School; 2 DDS. Associate Professor of Oral Surgery. Professor of the Master of Oral Surgery and Implantology. University of Barcelona Dental School. UB–IDIBELL Research group; 3 MD, DDS, PhD. Chairman of Oral and Maxillofacial Surgery. Director of the Master of Oral Surgery and Implantology. University of Barcelona Dental School. Oral and maxillofacial surgeon of the Teknon Medical Center, Barcelona (Spain). UB–IDIBELL Research group

## Abstract

Objectives: Assess the main problems referred by the patients and observed by the professionals after the bucodental rehabilitation with an implant-supported hybrid prothesis. Patient and Methods: A retrospective study was carried out in which there were 43 patients included who were visited in the Department of Oral Surgery and Orofacial Implantology of University of Barcelona Dental School for one year. An oral rehabilitation with an implant-supported hybrid prosthesis was made to those patients. The following variables were registered: age, gender, number of inserted implants, type of implant and principal problems produced by the hybrid prosthesis. Results: The rehabilitation with an implant supported hybrid prosthesis was only performed in 43 of 116 cases treated in one year (January, 2006 to January, 2007). They were 26 men and 17 women of ages between 37 and 74 years, being the rate age of 56,5 years. The main complication recorded was the mucositis, associated frequently with a difficulty to carry a correct oral hygiene and to an overextention of the tail of resin of the prosthesis. Other observed problems were the peri-implantitis, the break of the acrylic teeth and the loss of some of the prosthetic screws. Conclusions: The most frequent complication after the laying of an implant supported hybrid prosthesis was the mucositis, associated mainly with a prosthetic tail too long and to the consequent difficulty of carrying a correct oral hygiene. In spite of the high prevalence of observed complications, most of them were mild and resolved on subsequent visits.

** Key words:** Implant supported hybrid prosthesis, complications and prosthetic fails.

## Introduction

 The implant supported prostheses are becoming more used by the professionals to carry out oral rehabilitations (1,2) The concept of hybrid prosthesis is applied to any prosthesis that does not have a conventional design and that is normally composed by different types of materials. It can be fixed, removable or a Maxillofacial prosthesis (3). Generally we understand an hybrid prosthesis as the one which is composed by a substructure of noble metal that it is covered by acrylic teeth and that it is screwed on diverse implants. This way, it is a fixed prosthesis for the patient that can be removed by the professional when it is convenient (3). The hybrid prostheses can be made on a variable number of implants, with a minimum of 4, although there ideally should be placed the biggest number of implants that is possible (4).

This type of prosthesis has supposed a progress in the quality of life of edentulous patients compared with conventional complete dentures, since they offer functional, aesthetic and psychological advantages (1,2). Nevertheless, a series of mechanical, phonatory and infectious-inflammatory complications had been registered with the employment of this type of prosthesis. Between the above mentioned there are the mucositis and the peri-implantitis. The concept of mucositis alludes to an overextenion inflammatory reaction of reversible character, without bony loss, equivalent to the gingivitis of the periodontium. It is characterized principally by pain, gingival bled, erythema and ulcerations. When local harmful factors , mainly the plaque, perpetuate this inflammatory process may result in the loss of bone around the fixtures, being committed to the long-term rehabilitation.

In Jemt’s work (5) the main problems found were the break of the acrylic teeth and difficulties in the diction, both refered principally to the maxilla. On the other hand, he observed that the more frequent complications produced in the jaw were the injuries because of the lips and the cheeks bitting. 

Purcell et al. (6) valued the prosthetic complications that were produced after the laying a complete removable prosthesis in the maxilla and an hybrid mandibular prosthesis. The problems that affected the prosthetic fixed restoration were the break or the sweeping of the resin teeth and the loss, the wear or break of the prosthetic screw.

Authors as Carlson and Carlsson (7) found a wide fan of complications after the oral restoration with implant supported prostheses, whose resolution was going from the need to implement a small final touch to the dressmaking of a new prosthetic structure.

In the Goodacre’s et al. meta-analysis (8) the most frequent problem referred to the implant supported prosthesis was the break of the resin teeth. 

Nedir et al. (9) carried a comparison between the fixed prosthesis and the removable prosthesis on implants. They observed that the removable prosthesis were presenting a major number of complications than the fixed prosthesis and that these incidences were arising again later. 

The study of Aglietta et al. (10) reviewed the survival rates of fixed prostheses on implants with cantilever and the incidence of biological complications or those concerning the surgical technique after an observation period of 5 years.

The most prevalent problem with respect to the prosthesis were the fracture of teeth or loss of the prosthetic screw.

The objectives of this study were to assess the main problems reported by patients and observed by professionals after rehabilitation with an implant supported hybrid prosthesis in the Service of Oral Implantology of the Dental Clinic of the University of Barcelona.

## Patient and Methods

We present a retrospective study in which there were reviewed 116 medical records of patients that were visited and treated in the Service of Oral Implantology of the Dental Clinic of the University of Barcelona from January 2006 until January 2007. The sample includes 43 patients to whom a restoration with an implant supported hybrid prosthesis was carried. 26 men and 17 women aged between 37 and 74 years were treated , being the mean age of 56.5 years.

The hybrid prostheses was placed in 18 patients (41,9 %) on 6 implants and in 14 patients (32,5 %) on 4 implants. In some cases the dressmaking of the prostheses was carried out on 5, 7 or 8 implants ( Table 1).

The most used type of implant was that of parallel walls with external connection, principally Brånemark System® (Nobel Biocare AB, Gothenburg, Sweden) (44,2 %, n: 19) and Defcon TSH® (Impladent, Sentmenat, Spain) (34,8 %, n: 15). In 5 cases Nobel Replace was used® (Nobel Biocare, Gothenburg, Sweden) and in 3 Defcon TSA® (Impladent, Sentmenat, Spain). In a case there were used implants Dental Astra Tech Implant System® (Astra Tech AB, Mölndal, Sweden).

**Table 1:**
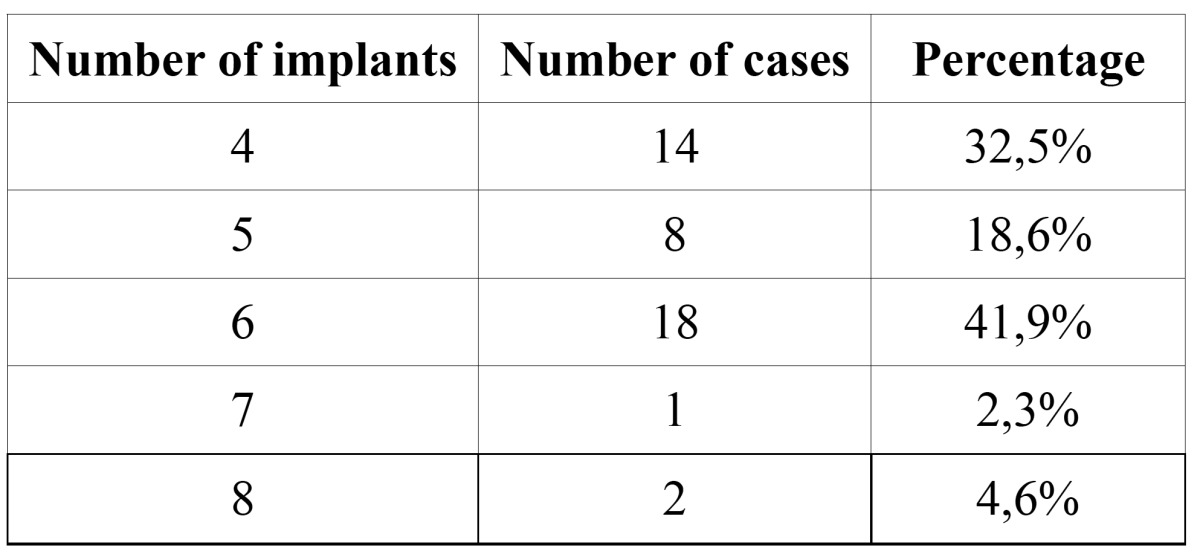
Number of implants placed in the cases included in the study.

The insertion of the implants and the dressmaking of the prosthesis was carried out by the residents of the Máster of Oral Surgery and Oral Implantology under the supervision of the professorship. 

The following variables were registered: the age, the gender, the number of inserted implants, the type of implant and the main problems produced by the hybrid prosthesis. A descriptive statistics was carried out by means of SPSS v15.0 of Windows (SPSS Inc., Chicago, USA).

## Results

The principal complication that registered was the mucositis, which affected 24 % of the cases (n: 12). The problems related to the prosthetic screw such as the break, the loss of the same one or the wear of the thread took place in 13,7 % of the cases (n: 7). With the same frequency there was observed the break of the teeth of the prosthesis or the sweeping of these and the peri-implantitis (13,7 %, n: 7). These problems were related to an incorrect record of the vertical dimension, an inadequate occlusion or to the absence of a passive adjustment of the metallic structure ( Table 2). 

Other problems that took place often was the fall of the material of obstruction of the chimneys of access to the prosthetic screw (7,8 %, n: 4). The same percentage of patients recounted difficulties on having carried out a correct hygiene of the prosthesis (7,8 %, n: 4). Most of the patients who recounted not to be able to carry out a good cleanliness of the prosthesis presented mucositis and an overextensión of the tail of resin of the prosthesis ( Table 2).

As for the distribution of dental arch complications, their prevalence was similar in the maxilla and the mandible (34.8%, n = 15 and 39.5%, n = 17, respectively) but noted that breakage or detachment from the teeth of the prosthesis was most often in the maxilla (18.6%, n = 8) than the lower (6.9%, n = 3), mainly by poor occlusal adjustment. Other complications observed in the mandible were the peri-implantitis and mucositis (11.6%, n: 5, each), while problems related to the prosthetic screw, fracture of the denture base and fall the filling material from the chimneys, were recorded each with a frequency of 4.6% (n: 2). Chewing problems or those arising from incorrect measurement of the vertical dimension occurred in 6.9% of cases (n = 3). With respect to the maxilla, mucositis and problems affecting the screw of the prosthesis occurred in 11.6% of cases each (n = 5), peri-implantitis, fracture of the base of the prosthesis, the fall of filling material from the chimneys of prosthetic screw access and biting injuries or problems resulting from poor fit of the vertical dimension was observed at 4.6% in each case (n = 2). In 3 of the patients complications took place in the maxilla and in the jaw simultaneously (6,9 %).

**Table 2:**
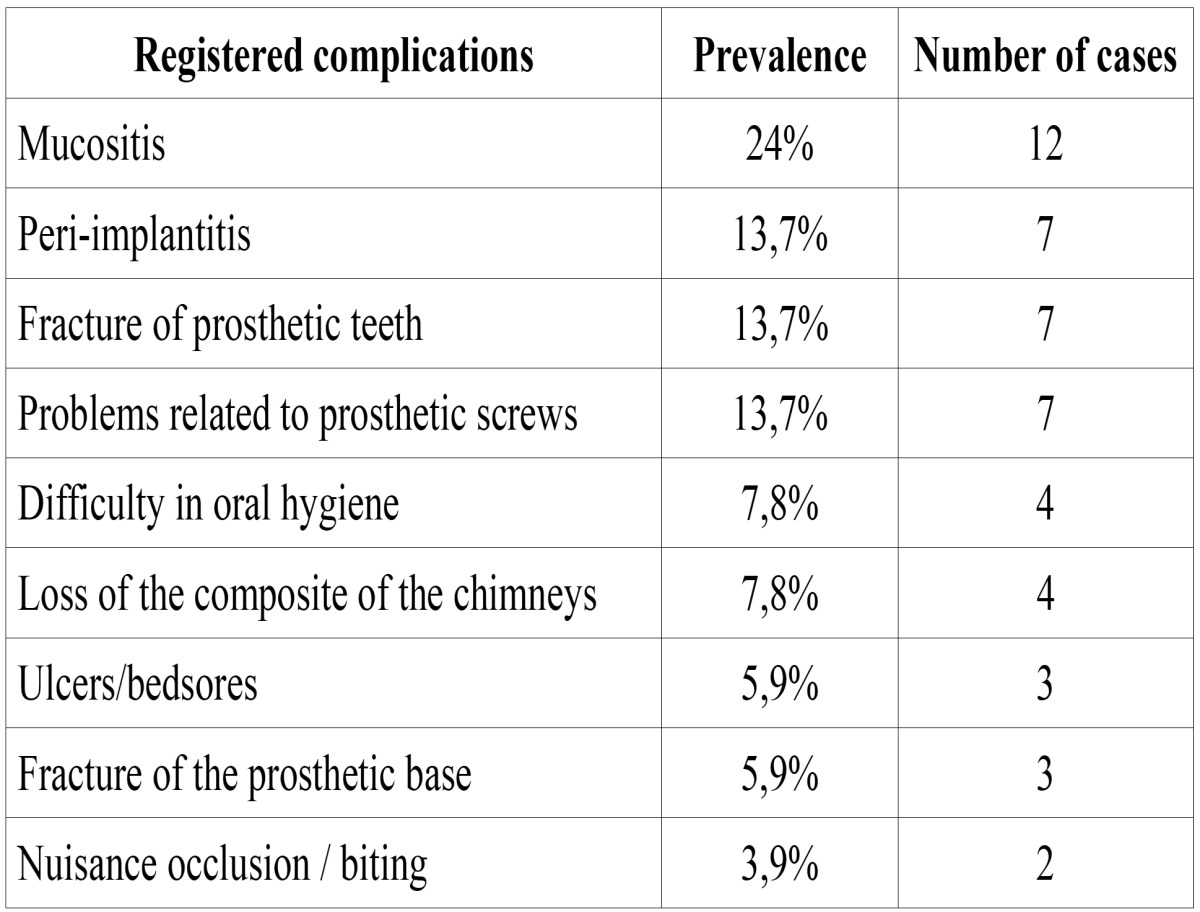
Frequency of complications in our patients.

**Figure 1:**
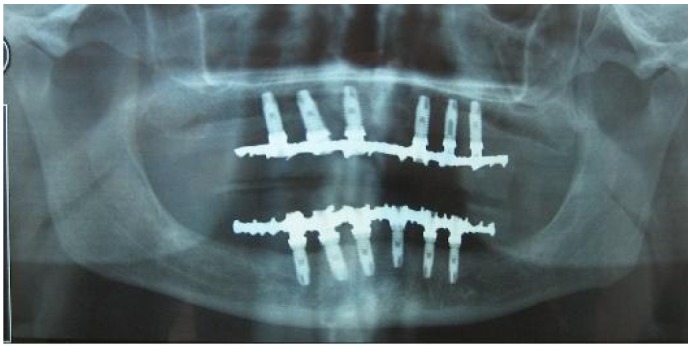
Orthopantomography of a 67-year-old patient rehabilitated with a superior hybrid prosthesis on 6 implants and a mandibular hybrid prosthesis on 6 implants.

**Figure 2:**
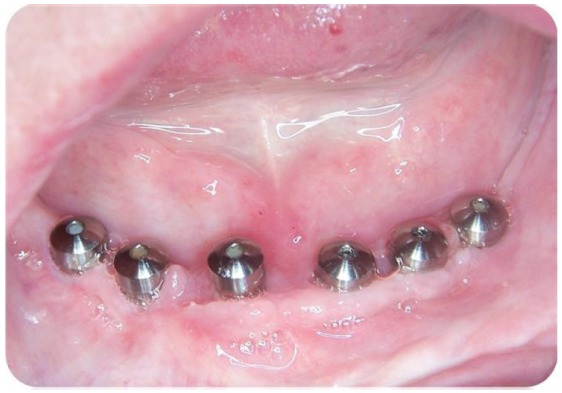
Initial clinical appearance. Shows the presence of mucositis around healing abutments.

**Figure 3:**
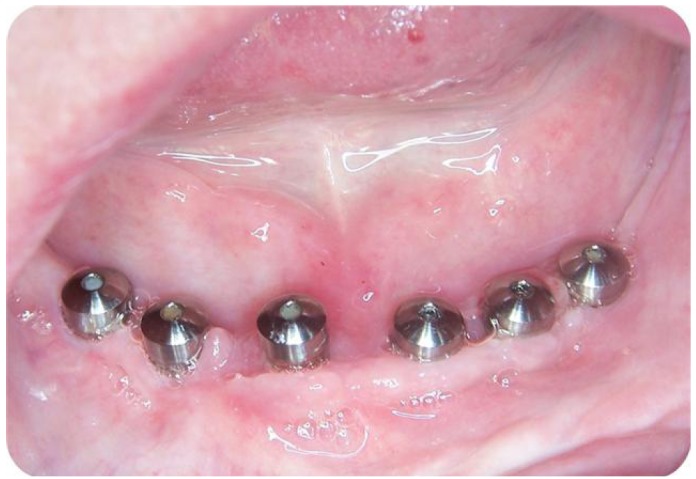
Picture clinic a week after treatment. Inflammation and gingival bleeding have decreased.

Some of the cases reviewed showed simultaneously a number of complications, as in the case presented in (Figs. 1, 2 and 3) for a 67-year-old patient who was rehabilitated with a prosthetic upper and lower hybrid prostheses on 6 implants in each jaw. Among the incidents that made this patient it is worth noting: injuries cheek biting, separation of upper lateral incisors and mucositis, the latter being more intense in the lower arch. The buccal mucosal injuries resolved spontaneously after a few days to adapt to the prosthesis, while the detachment of acrylic teeth was solved by making a careful occlusal adjustment. To correct mucositis hybrid prostheses were removed top and bottom and sanitized the area of the implants by curettage and irrigation of 0.12% chlorhexidine. The same mouthwash was prescribed to the patient to rinse made once every 12 hours for 15 days. We conducted a small flap cut acrylic prosthesis to facilitate the cleaning of it and instructed the patient to carry out proper oral hygiene. 

We recommend to use an irrigator, as well as of Superfloss (Oral B, Cincinnati, USA).

After treatment, the patient has not showed significant signs of gingival inflammation, however, recommended lifting the prostheses every 6 months to prevent possible complications.

As for the distribution of dental arch complications, their prevalence was similar in the maxilla and mandible, but found that the fracture or detachment from the teeth of the prosthesis was most often in the upper arch mainly by poor occlusal adjustment.

In relation to the moment of appearance of the complications, these took place in the days or the first weeks later to the laying of the prosthesis in mouth, except the mucositis and the peri-implantitis that they had a more late beginning. 

In 8 of 43 patients they did not register any type of complication (18,6 %). 15 patients presented simultaneously several of the problems previously mentioned (34,8 %). All complications were mild and resolved in the subsequent tests.

## Discussion

The prevalence of complications after the oral restoration with implant supported hybrid prostheses is high. However, there are few articles in the literature about this type of complications, making it difficult to compare results and assess whether the presence of prosthetic problems is frequent or not.

In 1991 Jemt (5) published a paper in which valued the presence of complications in implant prosthetics. After insertion of 2,199 implants in 391 edentulous jaws followed for one year he obtained a success rate of implants of 98.1%, with a success rate of prosthetic rehabilitation of 99.5%. He observed a higher percentage of problems in the maxilla than in the mandible. The main complications that he found in the upper jaw were the fracture of resin teeth and diction problems. In the mandible injuries took place for biting of the lips and the cheeks. All the complications were solved later. In our study there are no significant differences between the prevalence of the complications registered in the maxilla and the observed ones in the jaw. Nevertheless, we coincide with this author that the fracture or the detachment of the acrylic teeth is more frequent in the upper jaw. 

Purcell et al. (6) conducted a retrospective study that assessed the prosthetic complications appeared in 46 patients rehabilitated with complete removable upper denture and a mandibular hybrid prosthesis during a period of 5 years.

The most common complications were the fracture or the sweeping of the denture teeth, the need to refill the upper prosthesis and the problems related to the prosthetic screw.

Carlson and Carlsson (7) evaluated the complications following a dental restoration with osseointegrated implants. The range of complications reported was very wide, from the need to make a small adjustment to the preparation of a new prosthesis.The main problems occurred with the acrylic prosthesis. The prevalence of complications was higher in the maxilla than in the mandible. The loss of the implants was infrequent, occurred in only 1% of the examined patients. In our case, the principal complication that took place was the mucositis followed by the fracture of the resin teeth, the periimplantitis and the problems related to the prosthetic screw. Despite the prevalence of periimplantitis in our study (13.7%, n= 7), did not miss any implant. 

Aglietta et al. (10) determinated the survival rate of implant supported prostheses with cantilever and the incidence of technical or biological complications after a follow-up period of 5 years. They concluded that the survival rate was 94.3% and the complication rate was 88.9%. The most common complications were fracture of teeth or prosthetic screw loss followed by a decrease in the retention and fracture of prosthetic attachments.

Nedir et al. (9) conducted a comparison of fixed prostheses and removable prostheses on implants, concluding that the dentures had more complications than fixed, being the differences statistically significant. They noted that in the group treated with fixed prosthetic restorations complications occurred during the first two years after placement in the mouth and these were not recurring. Instead overdentures had repeatedly incidents and complications did not cease with the passage of time. In our study the complications related to the surgical technique are not rated. Neither the problems related to implant supported dentures. However, we agree with Nedir et al. (9) in which not enough studies in the literature on this topic to draw conclusions based on scientific evidence.

After the evaluation of cases in our study we think that the high prevalence of mucositis was due to improper oral hygiene, mainly produced by an overextension of the tail of hybrid denture resin. The term mucositis is usually used to describe a reversible inflammatory reaction without bone loss, equivalent to periodontal gingivitis. It is mainly characterized by pain, gingival bleeding, erythema and ulceration. The key to prevention is careful oral hygiene but can also be used surgical techniques to eliminate the hyperplasia of the surrounding soft tissue and keratinized gingival grafts in situations where it is necessary (11). When gingivitis is caused by poorly fitting dentures, as in our case, it is best to remove them and make the tweaks necessary to prevent the buildup of plaque. Can also be used antiseptic mouthwash to kill bacteria as well, to relieve the symptoms (12). The diode LASER can be used to 1.5-2W in the refractory cases to conventional treatment (11).

Peri-implantitis may have originated in the same way, by the accumulation of food and bacteria under the skirt acrylic prosthesis, but we also believe that in some cases intervened mechanical stress caused by a lack of passive fit of the metal structure or a malocclusion. In these cases the best treatment is to remove the prosthesis and through irrigation and curettage to remove the accumulated plaque. In cases in which there is evidence of infection will be necessary to resort to antibiotics. The antibiotic of choice is amoxicillin associated with clavulanic acid, although clindamycin and metronidazole are also indicated (11). If the marginal bone loss is important a guided bone regeneration technique can be indicated, although in none of our patients it has been necessary. We also associate the fracture of prosthetic screws to a mechanical factor. In those cases where it was observed that the lack of liability adjustment was responsible for the appearance of peri-implantitis or repeated fractures of the base of the prosthesis or the prosthetic screws,we had to make a new hybrid prosthesis, since it has been demonstrated that the appearance of these signs, a high overload produced for a parafunctional habit or a bad design in the location of the implants or the materials for making them, are the possible causes of fractures of the implants (13). In the study of Al Jabbari et al. (14) analyzed the causes of the fracture of the retention screws of implant supported prostheses in three patients. They observed with low-power stereomicroscope and high-power scanning electron microscopy the fractures produced in prosthetic screws, and they clearly showed a larger area of fatigue that coincided with the anterior media zone. These authors mention that the broken cracks screws can grow without the patient or the practitioner realize it, so it is difficult to solve the problem before rupture of the screw.

Regarding the fracture or detachment of resin teeth, we believe was caused by a bad adjustment of the occlusion or incorrect measurement of the vertical dimension. As for the loss of filling material from the chimneys of prosthetic screw access, it is produced by using a temporary material, in this case Fermit® (Ivoclar Vivadent, Schaan, Liechtenstein). This complication was resolved by changing the initial material for composite material (Spectrum® Dentsply, Mannheim, Germany). Lesions on cheeks and lips nibbling originated by a lack of adjustment to the new prosthesis, a fact that was resolved after several days of adaptation.

The results obtained in our study are similar to those found in the literature, except that for the majority of authors (5-8) the main complication was the fracture of acrylic teeth and for us was the mucositis. The broken teeth of the prosthesis or the detachment of these was the second most prevalent complication in our cases. Maybe if we were to extend the follow-up of our study results may change.

Despite the lack of bibliographic information related to this subject, it is necessary to reduce the prevalence of complications in implant prostheses in general and particularly in the hybrid prosthesis to further improve the quality of life for our patients. For this reason we believe that the characteristics that ideally should play a prosthesis of this type are, inter alia, prevent palatal coverage, provide adequate aesthetics and proper support to the lips and cheeks, to facilitate the maintenance of oral hygiene, the retention and phonetics are appropriate and that anchoring systems are functional and have an acceptable durability (15-17). It is also important to evaluate the interocclusal space, mainly in those patients with edentulous in both arches. This dimension will depend on the type of prosthetic restoration that is to be made, chose a hybrid prosthesis restoration when the space between the jaws is high.

Another important aspect to consider is the maintenance of prosthetic rehabilitation as well as the implants supporting the structure. Regular checks are recommended every 6 or 12 months to avoid complications and to assess the status of peri-implant tissue (18,19). Although studies, such as Lindsquist et al. (19), demonstrating that the success of fixed prostheses on long-term in edentulous patients in the mandible was 100%, other works such as Attard and Zarb (4) which was monitored 33 patients rehabilitated with fixed restorations in their edentulous jaws, reveal the need to perform maintenance since they observed that during the first 7 years, the success of implant prostheses was high (97.8%) and later they had to convert 6 fixed prostheses to overdentures because of the loss of several implants for not having the appropriate controls.

## Conclusions

The most common complication after placement of an implant supported hybrid prosthesis in our department was mucositis, mainly associated with prosthetic tail too long and therefore difficult to implement proper oral hygiene. Another complication that occurred with a high prevalence was the fracture of acrylic teeth, a finding consistent with that found in the literature.

It is important to carry out a correct record of the vertical dimension and to give a proper occlusion for each patient. We must also provide a passive adjustment to the metal frame of the prosthesis and make a prosthetic tail that offers, in addition to aesthetics, adequate access to facilitate oral hygiene.

We also believe that it is crucial to study the patient not only from a surgical point of view, but also prosthodontic as an indication of incorrect prosthesis can have an unacceptable level of complications.
